# Personalized medicine and the clinical laboratory

**DOI:** 10.1590/S1679-45082014RW2859

**Published:** 2014

**Authors:** João Renato Rebello Pinho, Roberta Sitnik, Cristóvão Luis Pitangueira Mangueira

**Affiliations:** 1 Hospital Israelita Albert Einstein, Sao Paulo, SP, Brazil.

**Keywords:** Molecular medicine, Genetics, medical, Individualized medicine, Neoplasms, DNA, Blood coagulation, Pharmacogenetics, Hepatitis C, Anticoagulants

## Abstract

Personalized medicine is the use of biomarkers, most of them molecular markers, for detection of specific genetic traits to guide various approaches for preventing and treating different conditions. The identification of several genes related to heredity, oncology and infectious diseases lead to the detection of genetic polymorphisms that are involved not only in different clinical progression of these diseases but also in variations in treatment response. Currently, it is possible to detect these polymorphisms using several methodologies: detection of single nucleotide polymorphisms using polymerase chain reaction methods; nucleic acid microarray detection; and nucleic acid sequencing with automatized DNA sequencers using Sanger-derived methods and new generation sequencing. Personalized medicine assays are directed towards detecting genetic variations that alter interactions of drugs with targets or the metabolic pathways of drugs (upstream and downstream) and can be utilized for the selection of drug formulations and detect different immunogenicities of the drug. Personalized medicine applications have already been described in different areas of Medicine and allow specific treatment approaches to be applied to each patient and pathology according to the results of these assays. The application of such a protocol demands an increasing interaction between the clinical laboratory and the clinical staff. For its implementation, a coordinated team composed of basic researchers and physicians highly specialized in their areas supported by a highly specialized team of clinical analysts particularly trained in molecular biology assays is necessary.

## INTRODUCTION

Personalized medicine is the use of biomarkers, most of them molecular markers, for the detection of specific genetic traits to guide different approaches for preventing and treating various pathologies. The identification of several genes related to heredity, oncology, and infectious diseases leads to the detection of genetic polymorphisms that are involved in different clinical progression of these diseases and in variations in treatment response.

Currently, it is possible to detect nucleic acid polymorphisms using several methodologies. Routine detection of single nucleotide polymorphisms (SNPs) can be achieved in clinical laborator ies using real time polymerase chain reaction (PCR). Other techniques, such as microarray detection or nucleic acid sequencing can also be routinely applied, especially when several different polymorphisms might be relevant to predict a particular genotype.^(1)^ New generation sequencing methodologies have now been incorporated into clinical laboratories, as they allow the simultaneous analysis of large regions of the genome and even the whole genome sequence, enabling the concurrent identification of different mutations in several genes.^(2,3)^

Personalized medicine assays are directed towards detecting genetic variations that alter interactions of drugs with targets or the metabolic pathways of drugs (upstream and downstream) and can be utilized for the selection of drug formulations and detection of diverse drug immunogenicities.^(4)^

The use of personalized medicine assays is broadening and several applications have already been described in different areas of medicine. Personalized medicine allows specific treatment approaches to be used for each patient and disease according to the results of these genetics assays. The application of such a protocol demands increasing interaction between the clinical laboratory and clinical staff. For its implementation, a coordinated team composed of basic researchers and physicians highly specialized in their areas supported by a highly qualified team of clinical analysts particularly trained in molecular biology assays is necessary.^(5)^

Some examples of clinical situations in which personalized medicine is already being used are detailed below.

## BREAST CANCER

For breast cancer, many different immunohistochemistry and molecular biology markers are utilized to indicate the most appropriate treatment option and to assess the risk of metastasis or recurrence.^(6)^

Treatment options include aromatase inhibitors (such as exemestane and anastrazole), indicated for adjuvant treatment of post-menopausal women with breast cancer positive for estrogen receptors (ER).^(7)^

Tamoxifen is the standard therapy for ER-positive breast cancer in pre-menopausal women.^(8)^ Proteins encoded by cytochrome P450 (CYP450) genes, CYP2D6 and CYP3A4, regulate the metabolism of tamoxifen. The efficacy of tamoxifen therapy is dependent on the CYP2D6 patient phenotype. More potent CYP2D6 inhibitors have had greater effects on plasma endoxifen concentrations, particularly antidepressants, such as paroxetine. Thus, knowledge of a drug’s ability to inhibit CYP2D6 enzyme activity may help clinicians anticipate clinically important drug interactions that would interfere with the metabolism of tamoxifen to its metabolites (N-desmethyltamoxifen, 4-hydroxytamoxifen, and endoxifen). The metabolite endoxifen maintains similar activity to N-desmethyltamoxifen and 4-hydroxytamoxifen but is found at higher levels in the plasma of patients receiving tamoxifen. Patients with the CYP2D6*1 allele maintain higher levels of the active metabolites. Patients with inherited variations in the CYP2D6 gene are poor tamoxifen metabolizers, resulting in diminished therapeutic activity and increased potential for adverse drug reactions (ADRs).^(9)^

High levels of HER-2/neu or ERBB2 (*v-erb-b2 erythroblastic leukemia viral oncogene homolog 2, neuro/glioblastoma-derived oncogene homolog*) expression have been associated with increased disease recurrence in breast cancer, but show a better response to trastuzumab.^(10)^

Finally, sequencing some genes, particularly BRCA (breast cancer) 1 and 2, guides surveillance and preventive treatment based on susceptibility risk for breast and ovarian cancers.^(11)^

## COLON CANCER

Colon cancer therapy and recurrence rates might also be accessed through genetic assays. Individuals who are homozygous for the UGT1A1 (*UDP glucuronosyltransferase 1 family, polypeptide A1*) *28 allele are at increased risk for neutropenia following initiation of irinotecan treatment, and a reduction in the starting dose should be considered for these patients.^(12)^

Non-mutated forms of BRAF (*v-raf murine sarcoma viral oncogene homolog B1) and *KRAS* (v-Ki-ras2 Kirsten rat sarcoma viral oncogene homolog*) genes are required for response to tyrosine kinase inhibitors (TKI).^(13)^ Immunohistochemistry studies with EGFR showed that patients with high expression are more likely to respond to these drugs than those with reduced expression.^(14)^

Panels of genes have also been developed to assess risk score or to guide therapy: (1) a seven-gene signature (plus five reference genes) provides a risk score that indicates whether a patient is likely to have tumor recurrence and guides the introduction of adjuvant therapy;^(15)^ (2) another assay used to guide therapy provides information about the expression of key molecular targets - KRAS, TS (*thymidylate synthase*), and TOP1 (*topoisomerase 1*);^(16)^ (3) expression profiles and mutations in ERCC1 (*excision repair cross-complementing rodent repair deficiency, complementation group 1*), TS, EGFR, BRAF, KRAS provide information for the selection of various therapies.^(16)^

Up to 30% of colorectal cancer has evidence of a familial component, and about 5% is associated with well-characterized inherited mutations. The sequencing of MLH1 (*mutL homolog 1*), MSH2 (*mutS homolog 2*), and MSH6 (*mutS homolog 6*) genes is recommended for surveillance and preventive treatment for the hereditary non-polypoid colon cancer or Lynch’s syndrome, which involves malignancies in other organs as well. There are also many other hereditary colorectal cancer syndromes, such as familial colorectal cancer type X, familial adenomatous polyposis, MUTYH-associated polyposis, Peutz-Jeghers syndrome, juvenile polyposis syndrome, PTEN (*phosphatase and tensin homolog gene*) hamartoma syndrome, and serrated polyposis syndrome.^(17)^

## LEUKEMIAS AND LYMPHOMAS

Philadelphia chromosome detection by fluorescent *in situ* hybridization (FISH) or the detection and quantification of the BCR/ABL (*breakpoint cluster region/c-abl oncogene 1, non-receptor tyrosine kinase*) gene translocation are routinely used in patients with chronic myeloid leukemia (CML)^(18)^ or acute lymphoblastic leukemia (ALL).^(19)^ Imatinib mesylate, dasatinib, or nilotinib are indicated for patients with BCR/ABL and the disease should be followed up by serial quantitative measures of the BCR/ABL.^(18)^ Patients treated with nilotinib who carry a UGT1A1*28 mutation have a high risk of hyperbilirubinemia.^(20)^

Arsenic trioxide and tretinoin are indicated for induction of remission and consolidation in patients with acute promyelocytic leukemia (APL), since this disease is characterized by the presence of t (15;17) translocation by FISH or PML/RARA (*promyelocytic leukemia/retinoic acid receptor, alpha*) expression detected by PCR.^(21)^

Patients with inherited poor or absent TPMT (*thiopurine S-methyltransferase*) activity are at increased risk for severe toxicity due to mercaptopurine, thioguanine, and azathioprine at conventional doses, and there is a recommendation for TPMT genotyping to adjust the dose for the treatment of ALL.^(22)^

Tositumomab is indicated for the treatment of patients with non-Hodgkin lymphoma expressing CD20 antigen.^(23)^ Denileukin difitox is indicated for the treatment of patients with persistent or recurrent cutaneous T-cell lymphoma whose malignant cells express the CD25 component of the interleukin 2 (IL-2) receptor.^(24)^

## LUNG CANCER

There are several genes involved in the selection of the most appropriate therapies for lung cancer. Expression profiles and mutations in ERCC1, TS, EGFR, RRM1 (*ribonucleotide reductase M1*), KRAS, and EML4-ALK (*echinoderm microtubule associated protein like 4 - anaplastic lymphoma receptor tyrosine kinase*) provide information for the selection of various treatments.^(16)^ Gemcitabine interferes with the DNA synthesis function of ribonucleotide reductase through its active subunit (RRM1), and low levels of RRM1 expression are associated with improved response to gemcitabine/carboplatin therapy.^(25)^ KRAS is mutated in about 30% of lung cancers, which exhibit resistance to EGFR-directed drugs (gefitinib and erlotinib) used in therapy. EGFR-activating mutations occur in approximately 10% of Caucasian patients with non-small cell lung cancer (NSCLC)and up to 50% of Asian patients. Data from multiple studies indicate a predictive role for EGFR-activating mutations with respect to response rate and progression-free survival with TKI therapy, particularly in the first-line setting.^(4,13)^ Crizotinib is indicated for the treatment of patients with locally advanced or metastatic NSCLC that is anaplastic lymphoma kinase (ALK)-positive, which occurs in 1 to 7% of these patients.^(26)^

## MELANOMA

Veramufenib is a kinase inhibitor indicated for the treatment of patients with unresectable or metastatic melanoma with an amino acid substitution from valine (V) to glutamic acid (E) at position 600 in BRAF. The BRAF V600E mutation is found in about half of melanoma patients.^(27)^

## MULTIPLE CANCERS

Tumors of unknown primary origin can be classified using gene expression profiles available in commercial or non-commercial assays.^(3,16)^

Tumor resistance to 5-fluorouracil (5-FU) is correlated with high levels of TS gene expression in colorectal tumors, primary adenocarcinoma of the stomach and lung cancer.^(28)^ Rarely, unexpected severe toxicity (*e.g.,* stomatitis, diarrhea, neutropenia, and neurotoxicity) associated with 5-fluorouracil has been attributed to a deficiency of dihydropyrimidine dehydrogenase (DPD) activity.^(29)^

Rasburicase administered to patients with glucose-6-phosphate dehydrogenase (G6PD) deficiency can cause severe hemolysis and patients should be screened prior to starting therapy, especially those at higher risk for G6PD deficiency, like patients of African or Mediterranean ancestry.^(30)^

ERCC1 helps repair DNA damage caused by platinum-based therapy: its low expression is associated with longer survival in advanced colorectal cancer treated with 5-fluorouracil/oxaliplatin while its high expression is associated with response to irinotecan therapy.^(31)^ Among gastric cancer patients, those treated with a FOLFOX (5-fluorouracil/leucovorin/oxaliplatin) regimen or first-line cisplatin-based regimens respond significantly better if they show lower levels of ERCC1 expression.^(32)^ Low ERCC1 is also a favorable indicator for response to platinum therapy in lung cancer.^(25)^

## PHARMACOGENOMICS

Pharmacogenomics studies the variability in response to drugs due to genetic variations. In the United States, the overall incidence of adverse reactions to drugs is about 6.7%, with about 0.3% of fatal accidents. Pharmacogenomics allows the precise treatment of a disease in each affected individual to improve drug efficacy and to reduce ADRs. ADRs are one of the most important causes of death in the United States with approximately 100 thousand deaths per year (fourth cause of death in the United States) and occur in 32% of hospitalized patients (with 6.7% of patients with serious but non-fatal ADRs). Besides the intrinsic and severe complications and the increased hospitalization times due to ADRs, they are also involved in increasing costs in the healthcare systems. In the United States, ADRs cost at least US$ 4 billion in annual direct costs of treatment, are responsible for 5% of hospital admissions, and increase the hospitalization length of stay, in average, by 2 days, increasing the cost approximately US$ 2,500 per patient.^(33,34)^

The chemical conversion of xenobiotics (drugs and other non-essential exogenous compounds) occurs mainly in the liver and leads to their conversion into hydrophilic substrates allowing urinary excretion. This metabolism can convert procarcinogenic compounds into cytotoxic and mutagenic ones. Pharmacogenomics studies the differences in metabolism between different individuals. It is important to emphasize that one xenobiotic metabolism may affect the metabolism of another and it is always relevant to know the complex and sometimes unexpected interactions among different drugs to better understand its pharmacokinetics in a specific individual.^(35)^

There are two phases in the metabolic pathway of xenobiotics. Phase 1 is called biotransformation and involves the attachment of functional groups and transformation of existing functional groups by oxidation, reduction, hydroxylation, hydrolysis etc. Phase 2 is called conjugation, *i.e.*, changing an existing functional group by acetylation, glycosylation, amino acid binding etc. These changes lead to a more hydrophilic compound, allowing its renal excretion.^(35)^

Phase 1 involves oxidations, such as the introduction of ketone, hydroxyl, or epoxide groups mediated by alcohol and aldehyde dehydrogenases, changing into amino groups by amino oxidases; alcohol conversions in aldehydes and acids by cytochrome P450 enzymes; reductions of ketones and nitro groups by nitro- and azoreductases; hydrolysis, *i.e.*, the breakdown of ester groups into alcohol and acid by esterases.^(35)^

Phase 2 involves changes in polar sites, such as by acetylation, amino acid conjugations, glucorinidation, methylation, and sulfation, catalyzed by different classes of transferases (*i.e.*, acetyl, glutathione, glucuronosyl, methyl, and sulfur).^(35)^

CYP450 is a large family of genes that encode liver enzymes responsible for drug and toxin metabolism. CYP450 involves seven different active genes, distributed into 17 families. CYP1, CYP2, and CYP3 are primarily involved in drug metabolism. CYP2A6, CYP2B6, CYP2C9, CYP2C19, CYP2D6, CYP2E1, and CYP3A4/5 are responsible for the metabolism of clinically important drugs. The most important reactions catalyzed by these enzymes are aliphatic oxidation, aromatic hydroxylation, sulfoxide formation, N-oxidation, N-hydroxylation, N-/O-/S- dealkylation, and oxidative or reductive dehalogenation.^(36)^

CYP2D6 and CYP2C19 enzymes metabolize approximately 25 to 30% of prescription drugs. CYP2D6 substrates include antiarrhythmics, antidepressants, beta-blockers, neuroleptics and other drugs, such as codeine, debrisoquine, phenformin, indoramin, and tamoxifen. The main drugs metabolized by CYP2C19 are amitriptyline, some barbiturates, clopidogrel, chlorproguanil, citalopram, cyclophosphamide, diazepam, imipramine, mephenytoin, and omeprazole.^(37)^

Each individual can be classified as one of the four types of drug metabolizers: (1) ultrarapid metabolizers (UM) carry multiple copies (three or more) of functional alleles and produce excessive enzymatic activities; (2) extensive metabolizers (EM) carry at least one normal functional allele; (3) intermediate metabolizers (IM) carry one reduced activity allele and one null allele or two reduced activity alleles; (4) poor metabolizers (PM) carry two mutant alleles which result in complete loss of enzyme activity.^(38-40)^

Genetic variants of these enzymes are found among different ethnic populations with a large variation in the genetic profile. These profiles have been determined for different populations around the world.^(41,42)^

Psychiatric drugs have many side effects that may cause interruption or failure of treatment. The time to optimum effect is long (3 to 8 weeks), requiring repeated dosages of serum for expensive tests that are also found in many clinical laboratories. Moreover, the selection and dosage of drugs is largely empirical and doses range from 5 to 20 times among different patients. Polymorphisms in CYP2D6 are one reason for the different individual responses as this enzyme metabolizes many of these drugs. For example, 7 to 10% of the Caucasian population did not have CYP2D6 activity (PM).^(43)^

Analysis of CYP2D6 gene dosage can guide therapy for depression. There are 45 million depressed people with nearly 15 million using medications. There are more than 400 thousand new cases of depression each year. In many countries, the first-line treatment is selective serotonin reuptake inhibitors (SSRIs), but 20 to 25% of patients do not respond to them and tricyclic antidepressants can be used as a second option for the treatment. Both classes of drugs are metabolized by CYP2D6. The CYP2D6 metabolizer profile for each patient has an important impact on time and costs of hospitalization, as generally the time spans needed for stabilizing drug dosage are longer in PM.^(44)^

Codeine and its derivatives are metabolized by CYP2D6 and are found in analgesics and antitussives, like oxycodone and hydrocodone. CYP2D6 genotyping is useful before starting therapy, since UM might experience dizziness, nausea, and agitation, while PM will have very limited benefits with codeine, but will not suffer the side effects or become chemically dependent on opioids.^(45,46)^

## HEPATITIS C

IL-28B gene polymorphisms have been reported as the strongest predictor of hepatitis C therapy response, early viral kinetics, and spontaneous viral clearance, and they might explain part of the association between therapy response and host ethnicity. Particularly in hepatitis C virus (HCV) genotype 1-infected patients, these SNPs (rs12979860 C/T, and rs8099917 T/G) have been shown to be highly associated with response to pegylated interferon/ribavirin treatment.^(47-49)^ In a study carried out in Brazil, IL28B rs12979860 and rs8099917 polymorphisms were predictors of therapy response not only in carriers of HCV genotype 1 but also in genotypes 2 and 3.^(50)^ With the new direct acting antivirals (DAAs) these results are not so relevant, although favorable genotypes are associated with better response levels to some shorter course treatments, and not all HCV infected patients are currently eligible for DAAs. IL28B polymorphism is still related to treatment outcomes, but the potent antiviral effect of these agents attenuates the strength of association. In some subgroups, as HIV/HCV-coinfected patients, pegylated interferon/ribavirin treatment will continue in use for most patients, and thus host factors will retain their predictive value for treatment outcomes for some time.^(51)^

Ribavirin-induced hemolytic anemia can complicate the management of patients treated for hepatitis C. Up to 15% of patients have to reduce ribavirin dosing due to severe anemia. A significant association was found between the hemoglobin drop in patients under antiviral therapy and inosine triphosphate (ITPA) deficiency associated with rs1127354 and rs7270101 polymorphisms in this gene.^(52)^ Reliable formulae for predicting the likelihood of ribavirin-induced anemia considering ITPA polymorphisms have been constructed that might be useful in developing individual optimization of ribavirin dosages to minimize drug-induced adverse events and further optimize anti-HCV treatment.^(53)^

## PLATELET ANTIAGGREGANTS

Clopidogrel is a platelet antiaggregant agent that has to be converted into its active metabolite (R 130694) by the action of different CYP450 isoforms, mainly CYP2C19. This prevents activation of the glycoprotein IIb/IIIa receptor complex, thus reducing platelet aggregation. This gene is highly polymorphic: CYP2C19*2, CYP2C19*3, CYP2C19*4, and CYP2C19*5 are inactive forms; CYP2C19*17 has increased activity. CYP2C19*2 and CYP2C19*3, inactive alleles, are frequent in the general population; CYP2C19*4 and CYP2C19*5 are rarer.^(54^^)^ An algorithm proposes that CYP2C19 genotyping testing should always be performed before or at the start of therapy. If the patient is a carrier of genotypes *1/*1, *1/*17, or *17/*17, standard dosing of clopidogrel can be utilized. On the other hand, if the patient is a carrier of at least one of the inactive alleles, prasugrel or another alternative therapy should be applied, as the expected effect of clopidogrel will not be present.^(55)^

## ANTICOAGULANTS

Warfarin is the most utilized anti-coagulant and it inhibits the regeneration of vitamin K epoxide to its reduced form, inhibiting synthesis of vitamin K-dependent clotting factors in the liver. Its full anticoagulant effect is manifested within 72 to 96 hours and its activity is monitored by the International Normalized Ratio (INR), with the goal of INR between 2 and 3. Warfarin is an enantiomeric mixture of equal concentrations of the R and S forms, mostly absorbed through the digestive tract. Warfarin is indicated in several syndromes in which thromboembolism might be present, such as atrial fibrillation, deep vein thrombosis, pulmonary embolism, stroke, and many others. The coagulation cascade requires vitamin K in the reduced form as a co-factor for gamma-glutamyl-carboxylase (GGCX) to convert inactive factors II, VII, IX, and X to the active form. Vitamin K is oxidized during this process to vitamin K epoxide. Warfarin inhibits the vitamin K epoxide reductase complex subunit 1 (VKORC1) that restores the available vitamin K to the reduced state, decreasing vitamin K availability, diminishing active factors II, VII, IX, and X, and thus inhibiting coagulation. The rate of metabolism of S-warfarin is approximately three times faster than for R-warfarin, and it is catabolized to its inactive metabolites S-6-OH-warfarin and S-7-OH-warfarin mainly by CYP2C9.^(56)^ Warfarin is a widely used anticoagulant with large inter-individual dose ranges and a narrow therapeutic index, as an insufficient dose may lead to thrombosis while an excessive dose can be associated with bleeding and hemorrhages. An increased bleeding risk for patients carrying either the CYP2C9*2 or CYP2C9*3 alleles has been shown. Certain single nucleotide polymorphisms in the VKORC1 gene (especially the 1639G>A allele) have been associated with lower dose requirements for warfarin.^(57)^ Different dosing algorithms have been proposed for warfarin considering age, sex, weight, or body surface area, race, use of other drugs (such as amiodarone, simvastatin, any azole), treatment indication (heart valve prosthesis, thromboembolic disease), INR, and VKORC1 (3673G>A) and CYP2C19 (*2, *3, *5) polymorphisms.^(57,58)^

## CONCLUSIONS

The approach for the patient in several areas of medicine is growing in complexity and an integrated vision of all the aspects involving each illness in each individual is now possible. Most of these advances have come from the progress obtained from a better and more comprehensive knowledge of the human genome during the last years. This increasing understanding of human genes has allowed predictions of how some mutations would generate clinical entities with diverse behaviors concerning their aggressiveness and treatment responses. The application of such tests is now routinely available, and the most frequent uses of this approach are described in the text.
