# Supplementary material for: Personalized medicine and the clinical laboratory
Source: Einstein (Sao Paulo). 2014 Jul-Sep;12(3):366–73. doi: 10.1590/S1679-45082014RW2859 (PMC4872953; doi:10.1590/S1679-45082014RW2859)
Supplement: Translation

# Translation: Medicina personalizada e o laboratório clínico

**Authors:** João Renato Rebello Pinho, Roberta Sitnik, Cristóvão Luis Pitangueira Mangueira

**Affiliations:** 1 Hospital Israelita Albert Einstein, São Paulo, SP, Brasil.

**Keywords:** Medicina molecular, Genética médica, Medicina individualizada, Neoplasias, DNA, Coagulação sanguínea, Farmacogenética, Hepatite C, Anticoagulantes

## Abstract

Medicina personalizada é o uso de biomarcadores, em sua maioria marcadores moleculares, para a detecção de traços genéticos específicos, a fim de orientar diversas abordagens para a prevenção e o tratamento de diferentes doenças. A identificação de vários genes relacionados a doenças hereditárias, oncológicas e infecciosas permite a detecção de polimorfismos genéticos que estão envolvidos em diferentes evoluções clínicas dessas doenças, bem como com variações na resposta ao tratamento. Atualmente, já é possível detectar esses polimorfismos utilizando diversas metodologias: a detecção de polimorfismos de nucleotídeo único pela reação de polimerização em cadeia; a detecção de microarranjos de ácidos nucleicos; e o sequenciamento de ácidos nucleicos com sequenciadores de DNA automatizados usando métodos derivados de sequenciamento Sanger ou de nova geração. Os ensaios de medicina personalizada são dirigidos para detectar variações genéticas que alteram interações de fármacos com alvos ou vias metabólicas de fármacos (anabólicas e catabólicas), podendo ser utilizados para a seleção de formulações farmacêuticas e para detectar diferentes imunogenicidades da droga. As aplicações de medicina personalizada já foram descritas em várias áreas da Medicina e permitem que abordagens de tratamento específicas sejam aplicadas para cada paciente e para cada doença, de acordo com os resultados dos ensaios utilizados. A aplicação de um protocolo desse tipo exige uma relação intensa entre o laboratório e o corpo clínico. Para sua execução, é necessária uma equipe coordenada, composta por investigadores de pesquisa básica e médicos altamente especializados em suas áreas, apoiada por um time bastante especializado de analistas clínicos treinados em testes de biologia molecular.

## INTRODUÇÃO

A medicina personalizada abrange o uso de biomarcadores, em sua maioria marcadores moleculares, para detectar traços genéticos específicos e orientar diferentes abordagens na prevenção e no tratamento de várias doenças. A identificação de vários genes relacionados a doenças hereditárias, oncológicas e infecciosas leva à detecção de polimorfismos genéticos, que estão envolvidos em diferentes evoluções clínicas dessas doenças e em variações nas respostas ao tratamento.

Atualmente, é possível detectar polimorfismos de ácidos nucleicos usando várias metodologias. A detecção rotineira de polimorfismos de nucleotídeos únicos (SNPs, sigla do inglês *single nucleotide polymorphisms*) pode ser conseguida em laboratórios clínicos usando a reação em cadeia da polimerase (PCR) em tempo real. Outras técnicas, como detecção de microarranjos ou sequenciamento de ácidos nucleicos, também podem ser rotineiramente aplicadas, especialmente quando vários polimorfismos diferentes podem ser relevantes na previsão de um genótipo em particular.^(1)^ Métodos de sequenciamento de nova geração têm sido incorporados em laboratórios clínicos, já que permitem a análise simultânea de amplas regiões do genoma e até da sequência inteira dele, viabilizando, assim, a identificação concomitante de diferentes mutações em vários genes.^(2,3)^

Os ensaios de medicina personalizada são focados na detecção de variações genéticas que alteram as interações de drogas com alvos ou as vias metabólicas das drogas *(*anabólicas e catabólicas* ou upstream* e *downstream*), podendo ser utilizados para a seleção de formulações de drogas e detecção de diversas imunogenicidades de drogas.^(4)^

O uso de ensaios de medicina personalizada está se ampliando e várias aplicações já foram descritas em diferentes áreas da medicina. A medicina personalizada permite que métodos terapêuticos específicos sejam usados para cada paciente e doença, conforme os resultados desses ensaios genéticos. A aplicação de tal protocolo demanda uma interação crescente entre o laboratório e a equipe clínica. Para sua implementação, é necessário contar com uma equipe coordenada, com pesquisadores de área básica e médicos altamente especializados em seus campos de estudo, com suporte de um time muito qualificado de analistas clínicos treinados, de forma específica, em ensaios de biologia molecular.^(5)^

Alguns exemplos de situações clínicas em que a medicina personalizada já está sendo utilizada são detalhados a seguir.

## CÂNCER DE MAMA

Para câncer de mama, diferentes marcadores imuno-histoquímicos e de biologia molecular são usados para indicar a opção de tratamento mais apropriada e avaliar os riscos de metástases ou recorrência.^(6)^

As opções de tratamento incluem inibidores de aromatase (como exemestano e anastrazol), indicados para o tratamento adjuvante de mulheres em pós-menopausa com câncer de mama positivo para receptores de estrógenos (ER).^(7)^

O uso do tamoxifeno é a terapia padrão para câncer de mama ER-positivo em mulheres na pré-menopausa.^(8)^ As proteínas codificadas por genes do citocromo P450 (CYP450), CYP2D6 e CYP3A4 regulam o metabolismo do tamoxifeno. A eficácia da terapia com tamoxifeno depende do fenótipo de CYP2D6 da paciente. Os inibidores mais potentes de CYP2D6 tiveram maiores efeitos sobre as concentrações plasmáticas de endoxifeno, particularmente os antidepressivos, como paroxetina. Assim, o conhecimento da capacidade de uma droga de inibir a atividade enzimática de CYP2D6 pode ajudar os clínicos a preverem interações medicamentosas clinicamente significantes, o que poderia interferir no metabolismo de tamoxifeno em seus metabólitos (N-desmetil-tamoxifeno, 4-hidroxi-tamoxifeno e endoxifeno). O metabólito endoxifeno mantém atividade similar às do N-desmetil-tamoxifeno e do 4-hidroxi-tamoxifeno, mas é encontrado em níveis mais elevados no plasma de pacientes que recebem o tamoxifeno. Pacientes com o alelo CYP2D6*1 mantêm níveis mais elevados dos metabólitos ativos. Pacientes com variações hereditárias no gene CYP2D6 são fracos metabolizadores de tamoxifeno, o que resulta na diminuição da atividade terapêutica e no aumento do potencial para reações adversas às drogas (RADs).^(9)^

Níveis de expressão elevados de HER-2/neu ou ERBB2 (v-erb-b2 *erythroblastic leukemia viral oncogene homolog 2, neuro/glioblastoma-derived oncogene homolog*) foram associados a um aumento de recorrência da doença no câncer de mama, mas demonstram melhor resposta ao trastuzumabe.^(10)^

Finalmente, o sequenciamento de alguns genes, particularmente de BRCA (*breast cancer*) 1 e 2, orienta a vigilância e o tratamento preventivo, baseado em risco de suscetibilidade para câncer da mama e do ovário.^(11)^

## CÂNCER DE CÓLON

A terapia do câncer de cólon e suas taxas de recorrência também podem ser avaliadas por meio de ensaios genéticos. Os indivíduos homozigóticos para UGT1A1 (*UDP glucuronosiltransferase 1, polipeptídio A1*) alelo *28 têm um risco aumentado para neutropenia após o início do tratamento com irinotecano e, nesses pacientes, deve ser levada em consideração uma redução na dose inicial.^(12)^

Formas não mutadas de genes de BRAF* (v-raf murine sarcoma viral oncogene homolog B1) *e KRAS *(v-Ki-ras2 Kirsten rat sarcoma viral oncogene homolog*) são necessárias para a resposta aos inibidores da tirosina quinase (TKI).^(13)^ Estudos de imuno-histoquímica com EGFR demonstraram que pacientes com alta expressão têm maior propensão a respostas a essas drogas do que aqueles com expressão reduzida.^(14)^

Também foram desenvolvidos painéis de genes para avaliar o escore de risco ou orientar a terapia: (1) uma assinatura de sete genes (mais cinco genes de referência) fornece um escore de risco que indica se um paciente possivelmente terá recorrência de um tumor e orienta a introdução de terapia adjuvante;^(15)^ (2) outro ensaio usado para orientar a terapia fornece informação sobre a expressão de alvos moleculares chaves - KRAS, TS (*thymidylate synthase*), e TOP1 (*topoisomerase 1*);^(16)^ (3) perfis de expressão e mutações em ERCC1 (*excision repair cross-complementing rodent repair deficiency, complementation group 1*), TS, EGFR, BRAF e KRAS fornecem informações para a seleção de várias terapias.^(16)^

Até 30% dos casos de câncer colorretal têm evidência de um componente familiar, e cerca de 5% estão associados a mutações hereditárias bem caracterizadas. O sequenciamento de genes de MLH1 (*mutL homolog 1*), MSH2 (*mutS homolog 2*) e MSH6 (*mutS homolog 6*) é recomendado para vigilância e tratamento preventivo do câncer não polipoide do cólon ou da síndrome de Lynch, que envolve doenças malignas em outros órgãos também. Igualmente, há muitas outras síndromes hereditárias de câncer colorretal, como o câncer colorretal familiar tipo X, a polipose familiar adenomatosa, a polipose associada a MUTYH, a síndrome de Peutz-Jeghers, a síndrome de polipose juvenil, a síndrome do hamartoma PTEN *(phosphatase and tensin homolog gene)* e a síndrome da polipose serrada.^(17)^

## LEUCEMIAS E LINFOMAS

A detecção do cromossomo Filadélfia por hibridização fluorescente *in situ* (FISH) ou a detecção e quantificação da translocação do gene BCR/ABL (*breakpoint cluster region/c-abl oncogene 1, non-receptor tyrosine kinase*) são usadas de rotina em pacientes com leucemia mieloide crônica (LMC)^(18)^ ou linfoblástica aguda (LLA).^(19)^ Mesilato de imatinibe, dasatinibe ou nilotinibe são indicados para pacientes com BCR/ABL, e a doença deve ser acompanhada por medidas quantitativas seriadas de BCR/ABL.^(18)^ Os pacientes tratados com nilotinibe que possuem a mutação UGT1A1*28 têm um risco alto de apresentar hiperbilirrubinemia.^(20)^

O trióxido de arsênico e a tretinoína são indicados para a indução da remissão e a consolidação em pacientes com leucemia promielocítica aguda (LPA), já que essa doença é caracterizada pela presença de translocação t (15;17) por FISH ou expressão PML/RARA (leucemia promielocítica/receptor de acido retinoico, alfa), detectada por PCR.^(21)^

Os pacientes com atividade hereditária pobre ou ausência de TPMT (tiopurina S-metiltransferase) apresentam um maior risco de toxicidade grave por mercaptopurina, tioguanina e azatioprina, em doses convencionais, e há uma recomendação de genotipagem de TPMT para ajustar a dose de tratamento da LLA.^(22)^

O tositumomabe é indicado para o tratamento de pacientes com linfoma não Hodgkin que expressa o antígeno CD20.^(23)^ Denileucina difitox é indicado para o tratamento de pacientes com linfoma cutâneo de células T persistente ou recorrente, cujas células malignas expressam o componente CD25 do receptor de interleucina 2 (IL-2).^(24)^

## CÂNCER DE PULMÃO

Existem vários genes envolvidos na seleção das terapias mais apropriadas para o câncer de pulmão. Os perfis de expressão e mutações em ERCC1, TS, EGFR, RRM1 (*ribonucleotídeo redutase M1*), KRAS e EML4-ALK (*echinoderm microtubule associated protein like 4 - anaplastic lymphoma receptor tyrosine kinase*) fornecem informações para a seleção de vários tratamentos.^(16)^ A gencitabina interfere na função de síntese de DNA da ribonucleotídeo redutase por meio de sua subunidade ativa (RRM1), e os baixos níveis de expressão de RRM1 estão associados a melhor resposta ao tratamento com gencitabina/carboplatina.^(25)^ KRAS é mutado em cerca de 30% dos casos de câncer de pulmão, que mostram resistência a drogas direcionadas a EGFR (gefitinibe e erlotinibe) usadas na terapia. As mutações ativadoras de EGFR ocorrem em aproximadamente 10% dos pacientes caucasianos com câncer de pulmão não pequenas células (CPCNP) e em até 50% dos pacientes asiáticos. Dados de múltiplos estudos indicam um papel preditivo para mutações ativadoras de EGFR com respeito à taxa de resposta e sobrevida livre de progressão com a terapia TKI, particularmente no contexto de primeira linha.^(4,13)^ O uso do crizotinibe é indicado para o tratamento de pacientes com CPCNP avançado localmente ou metastático, que seja positivo para quinase do linfoma anaplásico (ALK), que ocorre em 1 a 7% desses pacientes.^(26)^

## MELANOMA

O veramufenibe é um inibidor de quinase indicado para o tratamento de pacientes com melanoma irressecável ou metastático com uma substituição de aminoácido de valina (V) por acido glutâmico (E) na posição 600 de BRAF. A mutação BRAF V600E é encontrada em cerca de metade dos pacientes com melanoma.^(27)^

## MÚLTIPLOS TUMORES

Tumores de origem primária desconhecida podem ser classificados usando os perfis de expressão gênica disponíveis em ensaios comerciais ou não comerciais.^(3,16)^

A resistência tumoral ao 5-fluorouracil (5-FU) correlaciona-se a altos níveis de expressão do gene TS em tumores colorretais, adenocarcinoma primário do estômago e câncer do pulmão.^(28)^ Em casos raros, a toxicidade grave inesperada (por exemplo: estomatite, diarreia, neutropenia e neurotoxicidade) associada a 5-fluorouracil foi atribuída a uma deficiência da atividade de dihidropirimidina desidrogenase (DPD).^(29)^

A rasburicase administrada a pacientes com deficiência de glicose-6-fosfato desidrogenase(G6PD) pode causar hemólise grave, e os pacientes devem passar por uma triagem antes de iniciar a terapia, especialmente aqueles com alto risco de deficiência de G6PD, como os de ascendência africana ou mediterrânea.^(30)^

ERCC1 ajuda a reparar o dano ao DNA causado por terapia baseada na platina: sua baixa expressão está associada a uma sobrevida mais longa em câncer colorretal tratado com 5-fluorouracil/oxaliplatina, enquanto sua alta expressão está associada a resposta ao irinotecano.^(31)^ Dentre os pacientes com câncer gástrico, aqueles tratados com um esquema FOLFOX (5-fluorouracil/leucovorina/oxaliplatina) ou com esquema de primeira linha à base de cisplatina respondem de maneira significativamente melhor se mostrarem níveis mais baixos de expressão de ERCC1.^(32)^ Os níveis baixos de ERCC1 são também um indicador favorável de resposta à terapia com platina no câncer de pulmão.^(25)^

## FARMACOGENÔMICA

A farmacogenômica estuda a variabilidade de resposta a drogas em função de variações genéticas. Nos Estados Unidos, a incidência geral de RADs é de cerca de 6,7%, sendo 0,3% acidentes fatais. A farmacogenômica permite o tratamento preciso de uma doença em cada indivíduo afetado, a fim de melhorar a eficácia da droga e de reduzir as RADs. Tais reações representam uma das principais causas de morte nos Estados Unidos, com aproximadamente 100 mil mortes por ano (quarta causa de morte no país), e ocorrem em 32% dos pacientes hospitalizados (com 6,7% dos pacientes que apresentam RADs graves sérias, mas não fatais). Além das complicações intrínsecas e severas, e do tempo de hospitalização aumentado por causa das RADs, essas reações também estão envolvidas nos custos crescentes em sistemas de saúde. Nos Estados Unidos, as despesas com RADs são de, no mínimo, US$4 bilhões em custos diretos anuais de tratamento, representam 5% das internações, e aumentam o período de hospitalização, em média, em 2 dias, o que eleva o custo em aproximadamente US$2.500 por paciente.^(33,34)^

A conversão química de xenobióticos (drogas e outros compostos exógenos não essenciais) ocorre principalmente no fígado e leva à sua conversão para substratos hidrofílicos, o que permite sua excreção urinária. Esse metabolismo pode converter compostos pró-carcinogênicos em compostos citotóxicos e mutagênicos. A farmacogenômica estuda as diferenças de metabolismo entre diferentes indivíduos. É importante enfatizar que um metabolismo xenobiótico pode afetar o metabolismo de outro, sendo sempre relevante conhecer as interações complexas e, por vezes, inesperadas entre diferentes drogas, a fim de compreender melhor sua farmacocinética em um indivíduo específico.^(35)^

Há duas fases na via metabólica de xenobióticos. A fase 1 é chamada de biotransformação e envolve a fixação de grupos funcionais, além da transformação de grupos funcionais existentes pela oxidação, redução, hidroxilação, hidrólise etc. A fase 2 é chamada de conjugação, ou seja, a alteração de um grupo funcional existente pela acetilação, glicosilação, ligação a aminoácidos etc. Essas mudanças levam a um composto mais hidrofílico, permitindo sua excreção renal.^(35)^

A fase 1 envolve oxidações, como a introdução de grupos cetona, hidroxila ou epóxido mediada pelas desidrogenases de álcool e aldeído, levando à transformação em grupos de aminoácidos pelas amino oxidases; conversões de álcoois em aldeídos e ácidos pelas enzimas do CYP450; reduções de grupos cetona e nitro por nitrorredutases e azorredutases; hidrólise, isto é, a quebra de grupos éster em álcool em ácido pelas esterases.^(35)^

A fase 2 envolve alterações nos sítios polares, como por acetilação, conjugação de aminoácidos, glucorinidação, metilação e sulfatação, catalisados por diferentes classes de transferases (por exemplo: acetil, glutationa, glicuronosil, metil e enxofre).^(35)^

O CYP450 é uma grande família de genes que codificam enzimas hepáticas responsáveis pelo metabolismo de drogas e toxinas. O CYP450 envolve sete diferentes genes ativos, distribuídos em 17 famílias. CYP1, CYP2 e CYP3 estão envolvidos primariamente no metabolismo de drogas. CYP2A6, CYP2B6, CYP2C9, CYP2C19, CYP2D6, CYP2E1 e CYP3A4/5 são responsáveis pelo metabolismo de drogas clinicamente importantes. As reações mais importantes catalisadas por essas enzimas são oxidação alifática, hidroxilação aromática, formação de sulfóxidos, N-oxidação, N-hidroxilação, desalquilação N-/O-/S, e desalogenação oxidativa ou redutiva.^(36)^

As enzimas CYP2D6 e CYP2C19 metabolizam aproximadamente 25 a 30% das drogas prescritas. Os substratos de CYP2D6 incluem antiarrítmicos, antidepressivos, betabloqueadores, neurolépticos e outras drogas, como codeína, debrisoquina, fenformina, indoramina e tamoxifeno. As principais drogas metabolizadas por CYP2C19 são amitriptilina, alguns barbitúricos, clopidogrel, cloroproguanil, citalopram, ciclofosfamida, diazepam, imipramina, mefenitoína e omeprazol.^(37)^

Cada indivíduo pode ser classificado como sendo de um dos quatro tipos de metabolizadores de drogas: (1) metabolizadores ultrarrápidos (MU), que carregam múltiplas cópias (três ou mais) de alelos funcionais e produzem atividades enzimáticas excessivas; (2) metabolizadores extensivos (ME), que carregam pelo menos um alelo funcional ativo; (3) metabolizadores intermediários (MI), que carregam um alelo de atividade reduzida e um alelo nulo ou dois alelos de atividade reduzida; (4) metabolizadores pobres (MP), que carregam dois alelos mutantes, o que resulta em perda de atividade enzimática.^(38-40)^

As variantes genéticas dessas enzimas se encontram entre diferentes populações étnicas, com uma grande variação no perfil genético. Esses perfis já foram determinados para diferentes populações ao redor do mundo.^(41,42)^

As drogas psiquiátricas possuem muitos efeitos colaterais que podem causar interrupção ou falha de tratamento. O tempo para efeito ótimo é longo (3 a 8 semanas) e requer doses repetidas de soro para testes caros que também podem ser encontrados em muitos laboratórios clínicos. Ademais, a seleção e a dosagem de drogas são basicamente empíricas, e as doses variam de 5 a 20 vezes de um paciente a outro. Os polimorfismos de CYP2D6 são uma das razões para as diferentes respostas individuais, pois essa enzima metaboliza muitas dessas drogas. Por exemplo: de 7 a 10% da população branca não tinha atividade de CYP2D6 (MP).^(43)^

A análise da dosagem do gene CYP2D6 pode guiar o tratamento para depressão. Existem 45 milhões de pessoas deprimidas, sendo que cerca de 15 milhões usam medicamentos. São mais de 400 mil novos casos de depressão a cada ano. Em muitos países, o tratamento de primeira escolha são os inibidores seletivos de recaptação de serotonina (SSRIs), mas 20 a 25% dos pacientes não respondem a eles, e os antidepressivos tricíclicos podem ser usados como uma segunda opção. Ambas as classes terapêuticas são metabolizadas por CYP2D6. O perfil de metabolizador de CYP2D6, para cada paciente, tem um importante impacto sobre o tempo e os custos de hospitalização, já que geralmente os intervalos necessários para estabilizar a dosagem da droga são mais longos em MP.^(44)^

A codeína e seus derivados são metabolizados por CYP2D6 e são encontrados em analgésicos e antitussígenos, como oxicodona e hidrocodona. A genotipagem de CYP2D6 é útil antes de iniciar a terapia, já que os indivíduos MU podem sentir tonturas, náuseas e agitação, enquanto os MP têm benefícios muito limitados com a codeína, mas não sofrem os efeitos colaterais nem se tornam quimicamente dependentes de opioides.^(45,46)^

## HEPATITE C

A determinação de polimorfismos gênicos da IL-28B já foi descrita como a mais forte preditora de resposta à terapia para hepatite C, de cinética viral inicial, e de depuração viral espontânea e, possivelmente, ela explique parte da associação entre a resposta à terapia e a etnia do hospedeiro. Especialmente em pacientes infectados com o vírus da hepatite C (VHC) genótipo 1, esses SNPs (rs12979860 C/T, e rs8099917 T/G) já se mostraram muito associados à resposta ao tratamento com interferon pegilado/ribavirina.^(47-49)^ Em estudo realizado no Brasil, polimorfismos de IL28B rs12979860 e rs8099917 foram preditores da resposta à terapia não apenas em carregadores de VHC genótipo 1, mas também nos genótipos 2 e 3.^(50)^ Com os novos antivirais de ação direta (AADs), esses resultados não são tão relevantes, embora genótipos favoráveis estejam associados a melhores níveis de resposta a tratamentos de curta duração, e nem todos os pacientes infectados com VHC são atualmente elegíveis para receber AADs. O polimorfismo de IL28B está ainda relacionado a desfechos de tratamento, mas o potente efeito antiviral desses agentes atenua a potência da associação. Em alguns subgrupos, como o de pacientes coinfectados com HIV e VHC, o tratamento com interferon pegilado/ribavirina continua em uso para a maioria dos pacientes, e, assim, fatores do hospedeiro mantêm seu valor preditivo quanto a resultados do tratamento por algum tempo.^(51)^

A anemia hemolítica induzida por ribavirina pode complicar o cuidado de pacientes tratados para hepatite C. Até 15% dos pacientes precisam reduzir sua dosagem em função de anemia grave. Uma associação significante foi encontrada entra a queda em hemoglobina em pacientes sob terapia antiviral e deficiência de inosina trifosfato (ITPA) associada a polimorfismos rs1127354 e rs7270101 nesse gene.^(52)^ Já foram desenvolvidas fórmulas confiáveis para a predição da probabilidade de anemia induzida por ribavirina, considerando os polimorfismos de , que podem ser úteis no desenvolvimento da otimização individual de dosagens de ribavirina para minimizar os eventos adversos induzidos por drogas e otimizar ainda mais o tratamento anti-VHC.^(53)^

## ANTIAGREGANTES PLAQUETÁRIOS

Clopidogrel é um antiagregante plaquetário que tem de ser convertido para seu metabólito ativo (R 130694) pela ação de diferentes isoformas de CYP450, especialmente CYP2C19. Isso previne a ativação do complexo receptor glicoproteína IIb/IIIa, reduzindo, assim, a agregação plaquetária. Esse gene é altamente polimórfico: CYP2C19*2, CYP2C19*3, CYP2C19*4 e CYP2C19*5 são formas inativas; e CYP2C19*17 tem atividade aumentada. CYP2C19*2 e CYP2C19*3, alelos inativos, são frequentes na população geral; CYP2C19*4 e CYP2C19*5 são mais raros.^(54)^ Um algoritmo propõe que os testes de genotipagem de CYP2C19 sejam sempre feitos antes ou no início da terapia. Se o paciente é carregador de genótipos *1/*1, *1/*17 ou *17/*17, a dosagem padrão de clopidogrel pode ser utilizada. Por outro lado, se o paciente é portador de pelo menos um dos alelos inativos, prasugrel ou outra terapia alternativa deve ser aplicada, já que o efeito esperado do clopidogrel não estará presente.^(55)^

## ANTICOAGULANTES

A varfarina é o anticoagulante mais utilizado e inibe a regeneração do epóxido da vitamina K para sua forma reduzida, inibindo a síntese de fatores de coagulação no fígado que são dependentes de vitamina K. Seu pleno efeito anticoagulante é manifestado de 72 a 96 horas e sua atividade é monitorada pelo Índice de Normalização Internacional (INR), sendo o objetivo mantê-lo entre 2 e 3. A varfarina é uma mistura enantiomérica de concentrações iguais das formas R e S, a maior parte absorvida pelo trato digestivo. A varfarina está indicada em várias síndromes em que o tromboembolismo pode estar presente, como fibrilação atrial, trombose venosa profunda, embolia pulmonar, acidente vascular cerebral, e muitos outros. A cascata de coagulação requer vitamina K na forma reduzida como cofator para a gama-glutamil carboxilase (GGCX) converter fatores inativos II, VII, IX e X na forma ativa. A vitamina K é oxidada durante esse processo para epóxido de vitamina K. A varfarina inibe a subunidade 1 do complexo vitamina K epóxido redutase (VKORC1), que restaura a disponibilidade da vitamina K no estado reduzido, reduzindo a disponibilidade da vitamina K, diminuindo os fatores ativos II, VII, IX e X, e inibindo, portanto, a coagulação. A taxa de metabolismo de S-varfarina é aproximadamente três vezes mais rápida que para a R-varfarina, sendo catabolizada para seus metabólitos inativos S-6-OH-varfarina e S-7-OH-varfarina principalmente por meio de CYP2C9.^(56)^ A varfarina é um anticoagulante amplamente usado com grande variação de doses entre indivíduos e um índice terapêutico estreito, já que uma dose insuficiente pode levar à trombose, enquanto uma dose excessiva pode ser associada a sangramentos e hemorragia. Já foi demonstrado haver um risco aumentado de sangramento para pacientes que carregam os alelos CYP2C9*2 ou CYP2C9*3. Certos polimorfismos de nucleotídeo isolado no gene VKORC1 (especialmente o alelo 1639G>A) têm sido associados a necessidades de doses menores de varfarina.^(57)^ Diferentes algoritmos de doses têm sido propostos para a varfarina, levando em consideração idade, sexo, peso ou superfície corpórea, raça/etnia, uso de outras drogas (como amiodarona, sinvastatina e qualquer azol), indicação de tratamento (prótese de valva cardíaca e doença tromboembólica), INR, e polimorfismos de VKORC1 (3673G>A) e CYP2C19 (*2, *3, *5).^(57,58)^

## CONCLUSÃO

A abordagem para o paciente em várias áreas da medicina está crescendo em complexidade, e já é possível uma visão integrada de todos os aspectos que envolvem cada doença em cada indivíduo. A maioria desses avanços veio do progresso obtido por uma compreensão melhor e mais completa do genoma humano ao longo desses últimos anos. Esse conhecimento crescente dos genes humanos permitiu prever como algumas mutações iriam gerar doenças com comportamentos clínicos diferentes em relação à sua agressividade e resposta ao tratamento. A aplicação rotineira de tais testes atualmente é possível, e os usos mais frequentes dessa abordagem estão descritos no texto.
